# Dynamic Multiobjective Optimization Algorithm Based on Average Distance Linear Prediction Model

**DOI:** 10.1155/2014/389742

**Published:** 2014-01-30

**Authors:** Zhiyong Li, Hengyong Chen, Zhaoxin Xie, Chao Chen, Ahmed Sallam

**Affiliations:** ^1^College of Information Science and Engineering, Hunan University, Changsha 410082, China; ^2^College of Computer Science and Technology, Zhejiang University, Hangzhou 310027, China; ^3^Faculty of Computers and Informatics, Suez Canal University, Ismailia 41522, Egypt

## Abstract

Many real-world optimization problems involve objectives, constraints, and parameters which constantly change with time. Optimization in a changing environment is a challenging task, especially when multiple objectives are required to be optimized simultaneously. Nowadays the common way to solve dynamic multiobjective optimization problems (DMOPs) is to utilize history information to guide future search, but there is no common successful method to solve different DMOPs. In this paper, we define a kind of dynamic multiobjectives problem with translational Paretooptimal set (DMOP-TPS) and propose a new prediction model named ADLM for solving DMOP-TPS. We have tested and compared the proposed prediction model (ADLM) with three traditional prediction models on several classic DMOP-TPS test problems. The simulation results show that our proposed prediction model outperforms other prediction models for DMOP-TPS.

## 1. Introduction 

Due to the introduction of evolution algorithms, optimization algorithm research has got a great development, especially in the field of multiobjective optimization. However, in the last ten years, most of researchers focused on the stationary environment whose optimization process and evaluation functions are both clear and static. On the contrary, there are many important dynamic optimization problems in the real-world whose objective functions, constraints, and parameters may change with environment. These dynamic optimization problems with multiple objective functions are called dynamic multiobjective optimization problem (DMOP).

Multiobjective evolutionary algorithms (MOEAs) perform well for Multiobjective problems [1–3]; meanwhile they are adapted for DMOPs. Due to the dynamic characteristic, dynamic multiobjective evolutionary algorithms (DMOEAs) put more emphasis on the ability to track the new optimal front by coping with the environmental changes. There are mainly two ways to cope with changes in the environment: one way is to maintain diversity in MOEAs, which introduce new individuals when diversity of population decreases. Another way is to predict next Pareto solutions set using history information. Nowadays several successful prediction models have been proposed particularly for special problems; however, high-performance DMOEAs are the pursuits for solving DMOPs.

In this paper, when a change is detected in the dynamic environment, we study how to generate an initial population according to the own nature of the DMOP such that the closer the distance between initial population and the new Pareto set (PS) is, the more easily the new PS will be found. The initial population for the changed environment is generated by adding some new predicted individuals into current population; in this way the changed PS can be found more effectively by DMOEA. First, since the prediction model is relevant with the optimization problems, we define the DMOP-TPS problem whose PS translates regularly with time; then inspired by the nature of DMOP-TPS, the prediction strategies in [4, 5], and the strategy to generate an initial population in [6], we build a new prediction model to estimate the location of the new PS based on the information collected from previous search to solve DMOP-TPS. Finally, we test and compare our prediction model with other three superior prediction models on several DMOP-TPS test problems and we analyze the time complexity of the four prediction models. From the simulation results, it is obvious that our prediction model outperform the other three prediction models on DMOP-TPS. Moreover, from the time complexity analysis, it can be seen that the time complexity of ADLM is very low. In addition, we perform some interesting analysis on the simulation results about the efficiency of our proposed prediction model with particular problems.

## 2. Related Work

Recently, Evolutionary Dynamic Multiobjective Optimization (EDMO) has been intensively studied by many researchers. Benchmarks are famous for solving DMOPs through designing and testing relevant algorithms. In [7, 8], test problems are created by adding time-varying terms to the objectives in SMOPs. Yaochu Jin and Bernhard Sendhoff developed a method for constructing dynamic multiobjective test problems which clustered different objectives of existing stationary multiobjective problems (SMOPs) and changing the weights dynamically [9].

There are many ways to maintain population diversity; the simplest way is to reinitialize the population randomly [6]. Other common techniques include hypermutation [10], which increases the mutation rate when the improvement is not evident. Also, multiple population explorations can be treated as a diversity strategy; its basic idea is to use multiple populations to explore different regions, tracking the next optimal solutions [11–13]. Additionally, niche algorithm [14, 15] and crowding technique [16, 17] can be also applied in dynamic environment to maintain diversity. Although the diversity of population maintenance is necessary for DMOEA, most researchers maintained the diversity blindly rather than effectively when changes occur in the environment.

The method based on prediction model accelerates algorithms convergence. Zhou et al. used the Gaussian noise to update the current population, where the Gaussian parameters are determined by the historical changes [4]. Hatzakis and Wallace used the random time series forecasting model to evaluate the location of next optimal solutions, and then create individuals on the estimated location, which is used to seed the population when changes occur [5]. Since the prediction model is relevant to the optimization problems, we need to choose suitable prediction models to solve different problems. If the prediction is not precise, it may misguide the population to an incorrect region. Consequently, the convergence would slow down.

Arguably, diversity maintenance is essential in dynamic objective evolutionary optimization algorithms [18–20]; however, it is interesting to see that in multiobjective evolutionary algorithms, the diversity of population is inherently maintained due to the multiobjective nature. Accordingly, it is probably of greater importance to ensure that the population is able to follow the moving PS more quickly. To this end, a good guess of the new location of the changed PS is of great interest.

## 3. DMOP-TPS Problem

There are many kinds of DMOPs in the real-world, and different DMOPs have different natures. One of the differences between DMOPs is the changing rules of the PS, as the PS of some DMOPs changes regularly while the PS of others changes irregularly even randomly. In this section, we will define a special DMOP (DMOP-TPS), whose PS changes regularly with time.

### 3.1. Definition of DMOP

DMOP-TPS is a DMOP with special nature, so we will introduce the details of DMOP first.

**Definition 1 (DMOP [8])**

Let *t* be the time variable, **V** and **W** be the *n*-dimensional and *M*-dimensional continuous and discrete vector spaces, **g** and **h** be the two functions defining constraints inequalities and equalities, and **f** be a function from **V** × *t* to **W**. A dynamic multiobjective minimization problem with *M* objectives is defined as

(1) min⁡v∈Vs.t.f={f1(v,t),…,fM(v,t)},g(v,t)≤0, h(v,t)=0.

Like stationary MOP, we call the PS at time *t*(PS(*t*)) and the PF (Pareto front) at time *t*(PF(*t*)) of the set of Paretooptimal solutions at time *t* in decision variable and objective spaces, respectively; therefore, there are four possible ways for a problem to demonstrate a time-varying change [7] as follows.

The PS changes, whereas the PF does not change.

1. Both PS and PF change.
2. PS does not change, whereas the PF changes.
3. Both PS and PF do not change, whereas the problem can change.

It is a possible that while the problem changes, more types of above changes can occur simultaneously in the time scale.

### 3.2. Definition of DMOP-TPS

Now, the definition of DMOP-TPS could be given as follows.

**Definition 2 (DMOP-TPS)**

PS(*t*) and PS(*t* + 1) are the Paretooptimal set of DMOP at time *t* and *t* + 1, respectively; *A*(*t*) = (*a*_1_(*t*), *a*_2_(*t*),…, *a*_*n*_(*t*)) is *n*-dimensional vector. A DMOP is a DMOP-TPS if and only if for any decision variable *X* ^*t*^ = (*x*_1_^*t*^, *x*_2_^*t*^,…, *x*_*n*_^*t*^) ∈ PS(*t*), there must be a decision variable *X* ^*t*+1^ = (*x*_1_^*t*+1^, *x*_2_^*t*+1^,…, *x*_*n*_^*t*+1^) ∈ PS(*t* + 1), which satisfies the constraints {*x*_1_^*t*+1^ = *x*_1_^*t*^ + *a*_1_(*t*), *x*_2_^*t*+1^ = *x*_2_^*t*^ + *a*_2_(*t*),…, *x*_*n*_^*t*+1^ = *x*_*n*_^*t*^ + *a*_*n*_(*t*)}.

From the intuitive point of view, DMOP-TPS is a kind of DMOP with special nature whose PS translates regularly with time.

## 4. Dynamic Multiobjectives Evolutionary Algorithm with ADLM Model (DMOEA/ADLM)

DMOEA is the mainstream for solving DMOP, and it was built based on the static MOEA. However, in order to effectively solve DMOP, DMOEA built its own framework because of the dynamic property of DMOP. Also, some operators coping with environmental changes have been added, where the prediction is one of these operators.

### 4.1. The Framework of DMOEA with Prediction Model (DMOEA/PRI)

The main steps of the dynamic multiobjective evolutionary algorithm with prediction model could be described as in Algorithm 1.

In this paper, we focus on the population reinitialization once a change is detected in the environment. In the following, other operators are briefly introduced.

To detect the environmental change, we take the strategy proposed by Deb et al. [6] to recalculate the function values of some individuals selected from current population. If their objective values change, then the environmental change will occur. In (8), *ζ*% of *P*_*τ*_ is replaced with randomly selected solutions from PD_*k*_ to generate a reinitialized population with size *N* when the environment changed; otherwise, a new population is generated by reserving *N* better individuals from the union set of current population *P* and the offspring population *P*_*τ*_ in (12). The population size maintains *N* in each iteration.

In [1], Deb et al. have proposed the famous algorithms (NSGA-II) to tackle static multiobjective optimization problems. By taking into account the regularity property of MOPs, NSGA-II can approximate the PF efficiently. In this paper, the operators in NSGA-II are used in (11) of the above framework; its basic idea is to use SBX crossover operator and polynomial mutation operator to generate offspring. Nondominated sorting of NSGA-II is used in (12) to select better solutions to be retained. The details of (4) will be discussed in the following subsection.

### 4.2. General Prediction Model

To insure faster convergence of the new PF when a change is detected in the environment, it is a good choice to predict the new locations of the Paretooptimal solutions with historical information. We assume that *Q*_*t*_,…, *Q*_1_ can provide enough information for predicting the new location of PS_*t*+1_ and the location of PS_*t*+1_ is a function of the locations *Q*_*t*_,…, *Q*_1_:

(2) $$\begin{array}{c} {\text{PD}}_{t}=F\left(Q_{t},\ldots ,Q_{1},t\right). \end{array}$$

The prediction now becomes how to use the historical information *Q*_*t*_,…, *Q*_1_ to generate new individuals PD_*t*_ which should be close enough to PS_*t*+1_.

In practice, it is very hard to build a general prediction model for all kinds of problems. Sometimes, a prediction model performs well on one kind of problem and performs poorly on another one. This is because the changing rule of the PS(*t*) is unknown and not consistent for different kinds of problems. Therefore, if one prediction model is designed for a specific kind of problem, the accuracy of the prediction model will be improved obviously. In the following, we discuss how to generate initial solutions for time window *t* + 1.

Suppose that *X*_*t*_, *X*_*t*−1_,…, *X*_1_  (*X*_*i*_ ∈ *Q*_*i*_, *i* = *t*,…, 1) are a set of individuals in the decision space which describes the movements of the PS. A generic model that predicts the location of the initial individuals for the (*t* + 1)th time window could be formulated as follows:

(3) $$\begin{array}{c} X_{t+1}=F\left(X_{t},X_{t-1},\ldots ,X_{t-k+1},t\right). \end{array}$$

For one individual *X*_*t*_ ∈ *Q*_*t*_, the location of its parent in the previous time window could be defined as the nearest individual in  *Q*_*t*−1_; that is,

(4) $$\begin{array}{c} X_{t-1}=\arg\underset{X\in Q_{t-1}}{\min}{||X-X_{t}||}_{2}. \end{array}$$

Any time series models [21] could be used for modeling *F* in (3).

Any linear or nonlinear prediction model could be used to predict the location of the individual for the next time window, when time series are identified for each individual in the population.

### 4.3. The New Prediction Model (ADLM)

One reasonable and accurate prediction model is always designed according to the special nature of DMOP. This way guarantees that the prediction model will be suitable to solve corresponding DMOP. Since the proposed ADLM is designed based on the nature of DMOP-TPS, the mathematical properties of DMOP-TPS will be introduced first.

#### 4.3.1. Mathematical Properties of DMOP-TPS

Assume that *X* ^*t*^ is a Paretooptimal solution of DMOP-TPS, if *X* ^*t*^ translates with time *t* according to a continuous differentiable function, then we get mathematical properties of DMOP-TPS as Theorem 3.

**Theorem 3**

*f*(*t*) is a continuous differentiable function of time *t*, *t*_1_, *t*_2_, *t*_3_, and *t*_4_ are four adjacent time, and Δ*t* is the interval between two adjacent time; if Δ*t* → 0, then the following equation is accordant:

(5) $$\begin{array}{c} f\left(t_{4}\right)=f\left(t_{3}\right)+\left|\frac{f\left(t_{3}\right)-f\left(t_{2}\right)}{f\left(t_{2}\right)-f\left(t_{1}\right)}\right|\times \left(f\left(t_{3}\right)-f\left(t_{2}\right)\right). \end{array}$$

**Proof**

*f*(*t*) is continuous and differentiable, so the derivative of *f*(*t*), which is marked as *f*′(*t*), is also continuous; that means that the interval between *f*′(*t*_1_), *f*′(*t*_2_), *f*′(*t*_3_),  and*f*′(*t*_4_) will become smaller when Δ*t* → 0; thus, the following equation is true:

(6) $$\begin{array}{c} f^{\prime }\left(t_{3}\right)=\left|\frac{f^{\prime }\left(t_{2}\right)}{f^{\prime }\left(t_{1}\right)}\right|\times f^{\prime }\left(t_{1}\right),\\ f^{\prime }\left(t\right)=\frac{f\left(t+\Delta t\right)-f\left(t\right)}{\Delta t}. \end{array}$$

Combining the above two equations, we get

(7) f(t3+Δt)−f(t3)Δt =|(f(t2+Δt)−f(t2))/Δt(f(t1+Δt)−f(t1))/Δt|×f(t2+Δt)−f(t2)Δt.

Multiplying (7) with Δ*t*, then we get

(8) f(t3+Δt)−f(t3) =|f(t2+Δt)−f(t2)f(t1+Δt)−f(t1)|×(f(t2+Δt)−f(t2)).

Based on function *f*(*t*_*k*+1_) = *f*(*t*_*k*_ + Δ*t*), (8) could be simplified to

(9) $$\begin{array}{c} f\left(t_{4}\right)-f\left(t_{3}\right)=\left|\frac{f\left(t_{3}\right)-f\left(t_{2}\right)}{f\left(t_{2}\right)-f\left(t_{1}\right)}\right|\times \left(f\left(t_{3}\right)-f\left(t_{2}\right)\right). \end{array}$$

Equation (9) is equal to (5); thus, the proof is completed.

#### 4.3.2. ADLM

In this paper, in order to solve DMOP-TPS, the following linear model which is designed according to the mathematical properties of DMOP-TPS will be adopted and hereafter will be called ADLM for short:

(10) $$\begin{array}{c} X_{t+1}=X_{t}+\text{Gaussian}\left(\text{mean}\left(t\right),v\right)\times \left(\bar{X_{t}}-\bar{X_{t-1}}\right),\\ \text{mean}\left(t\right)=\left|\frac{\bar{X_{t}}-\bar{X_{t-1}}}{\bar{X_{t-1}}-\bar{X_{t-2}}}\right|. \end{array}$$

Gaussion(mean(*t*), *v*) is Gaussian random distribution function. $\bar{X_{t}}-\bar{X_{t-1}}$ is the distance between average *X*_*t*_ ∈ *Q*_*t*_ and average *X*_*t*−1_ ∈ *Q*_*t*−1_; likewise, $\bar{X_{t-1}}-\bar{X_{t-2}}$ is the distance between average *X*_*t*−1_ ∈ *Q*_*t*−1_ and average *X*_*t*−2_ ∈ *Q*_*t*−2_.

Apparently, mean(*t*) and $\bar{X_{t}}-\bar{X_{t-1}}$ are corresponding to |(*f*(*t*_3_) − *f*(*t*_2_))/(*f*(*t*_2_) − *f*(*t*_1_))| and *f*(*t*_3_) − *f*(*t*_2_) in (5), respectively. Therefore, ADLM is reasonable and accurate as a prediction model for solving DMOP-TPS problem.

## 5. Experiments on DMOP-TPS

This section is devoted to present the experiments performed in this work. First, we introduce the set of MOPs used as a benchmark. Next, we describe the indicators applied for measuring the performance of the obtained PF. Then, we list the prediction models for comparison. Finally, we present the comparisons of the four prediction models.

### 5.1. Benchmarks

To examine the performance of our proposed algorithm (DOMEA/ADLM) on DMOP-TPS, we have used six test benchmarks in our simulation studies. The first three test problems are FDA1 [7], FDA1E, and FDA1L:

(11) $$\begin{array}{c} f_{1}\left(X_{Ι}\right)=x_{1},\\ g\left(X_{ΙΙ}\right)=1+\sum_{x_{i}\in X_{ΙΙ}}{\left(x_{i}-G\left(t\right)\right)}^{2},\\ h\left(f_{1},g\right)=1-\sqrt{\frac{f_{1}}{g}},\\ G\left(t\right)=\sin\left(0.5\pi t\right),\;t=\frac{1}{n_{t}}\left\lfloor \frac{\tau }{\tau_{T}}\right\rfloor ,\\ X_{Ι}=\left(x_{1}\right)\in \left[0,1\right],\;\;X_{ΙΙ}=\left\{x_{2},\ldots ,x_{n}\right\}\in \left[-1,1\right]. \end{array}$$

Formula (11) represents the FDA1 problem, while FDA1E and FDA1L are the exponential and logarithmic style of FDA1 are respectively. The FDA1E and FDA1L are as follows

(12) $$\begin{array}{c} f_{1}\left(X_{Ι}\right)=x_{1},\\ g\left(X_{ΙΙ}\right)=1+\sum_{x_{i}\in X_{ΙΙ}}{\left(x_{i}-G\left(t\right)\right)}^{2},\\ h\left(f_{1},g\right)=1-\sqrt{\frac{f_{1}}{g}},\\ G\left(t\right)=2^{t}-1,\;t=\frac{1}{n_{t}}\left\lfloor \frac{\tau }{\tau_{T}}\right\rfloor ,\\ X_{Ι}=\left(x_{1}\right)\in \left[0,1\right],\;\;X_{ΙΙ}=\left\{x_{2},\ldots ,x_{n}\right\}\in \left[-1,1\right],\\ f_{1}\left(X_{Ι}\right)=x_{1},\\ g\left(X_{ΙΙ}\right)=1+\sum_{x_{i}\in X_{ΙΙ}}{\left(x_{i}-G\left(t\right)\right)}^{2},\\ h\left(f_{1},g\right)=1-\sqrt{\frac{f_{1}}{g}},\\ G\left(t\right)=\text{lo}{\text{g}}^{t+1},\;t=\frac{1}{n_{t}}\left\lfloor \frac{\tau }{\tau_{T}}\right\rfloor ,\\ X_{Ι}=\left(x_{1}\right)\in \left[0,1\right],\;\;X_{ΙΙ}=\left\{x_{2},\ldots ,x_{n}\right\}\in \left[-1,1\right]. \end{array}$$

The second three test problems are FDA5 [7], FDA5E, and FDA5L:

(13) min⁡X f1(X)=(1+g(XΙΙ))∏i=1M−1cos⁡⁡(yiπ2),min⁡Xfk(X)=(1+g(XΙΙ))(∏i=1M−kcos⁡⁡(yiπ2)  ) ×sin(yM−k+1π2)      k=2,…,(M−1),min⁡XfM(X)=(1+g(XΙΙ))sin(y1π2),whereg(XΙΙ)=G(t)+∑xi∈XΙΙ(xi−G(t))2,yi=xiF(t) for    i=1,…,(M−1),G(t)=|sin⁡(0.5πt)|,F(t)=1+100  sin4(0.5πt),t=1nT⌊ττT⌋,XΙΙ=(xM,…,xn),   xi∈[0,1],i=1,…,n.

Formula (13) represents the FDA5 problem, while FDA5E and FDA5L are the exponential and logarithmic style of FDA5, respectively.

In all the six test problems, *τ* is the generation counter, *τ*_*T*_ is the number of generations in each time window, and *n*_*T*_ controls the distance between two consecutive PS (the bigger the *n*_*T*_ is, the smaller the distance is). In fact, *τ*_*T*_ and *n*_*T*_ represent the frequency of change, and severity of change respectively.

FAD1, FAD1E, FAD1L, FAD5, FAD5E, and FAD5L are all DMOP-TPS problems; the first three belong to the DMOP problems of (I) and the second three belong to the DMOP problems of (II).

Above all, as test problems, the six DMOPs are comparatively comprehensive for testing the performance of DMOEA.

### 5.2. Prediction Models for Comparison

In this experiment, four prediction models will be tested; they are MM, VARM, PREM, and ADLM; all prediction models are listed as follows besides ADLM.

(1) MM: MM is a very simple prediction model [6]; it generates new individuals based on all individuals *X*_*t*_ ∈ *Q*_*t*_ by polynomial mutation operator; that is,
  (14) $$\begin{array}{c} X_{t+1}=\text{mutation}\left(X_{t}\right). \end{array}$$
(2) VARM: the model of VARM [5] is listed as follows
  (15) $$\begin{array}{c} X_{t+1}=X_{t}+\varepsilon , \end{array}$$
  where *ε* is a normal distribution; that is,
  (16) $$\begin{array}{c} \varepsilon \sim N\left(0,\delta I\right), \end{array}$$
  where *I* is an identity matrix and *δ* is the standard deviation, which is defined by
  (17) $$\begin{array}{c} \delta^{2}=\frac{1}{4n}{||X_{t}-X_{t-1}||}_{2}^{2},\\ X_{t-1}=\arg\underset{X\in Q_{t-1}}{\min}{||X-X_{t}||}_{2}. \end{array}$$
(3) PREM: the model of PREM [5] is listed as follows:
  (18) $$\begin{array}{c} X_{t+1}=X_{t}+\left(X_{t}-X_{t-1}\right)+\varepsilon , \end{array}$$
  where *ε* is the same with the *ε* in VARM.

### 5.3. Performance Indicators

In this paper, a distance-based performance indicator *D*(*P*) suggested in [3] is used to measure the convergence of DMOEA:

(19) $$\begin{array}{c} M\left(P\right)=D\left(P\right)=\frac{1}{\left|P^{*}\right|}\sum_{x\in P^{*}}{||x-y\left(x\right)||}_{2}, \end{array}$$

where *P* is an obtained nondominated set, P* is a reference PF, and

(20) $$\begin{array}{c} y\left(x\right)=\arg\underset{y\in P}{\min}{||x-y||}_{2}. \end{array}$$

Let *M*(*P*_*t*_) measure the performance (the smaller, the better) of population *P* at time *t*; to assess the performance of an algorithm fairly, we record the following averages of the means over *t* in our experiments:

(21) $$\begin{array}{c} \text{Ave}\left(M\left(P\right)\right)=\frac{1}{T}\sum_{t=1}^{T}M\left(P_{t}\right). \end{array}$$

Supposing an algorithm runs *N* times on a given problem, we use

(22) $$\begin{array}{c} \text{Ave}\left(M\left(P\right)\right)=\frac{1}{T\times N}\sum_{t=1}^{T}\sum_{i=1}^{N}M\left(P_{t}^{i}\right), \end{array}$$

to denote the mean of the performance indicator *M* at all time in all runs, which is inspired by the idea of the offline error metric [20].

### 5.4. Results and Discussion

In our experiments, the number of dimensions of the decision variables is 30 for FDA1, FDA1E, and FDA1L and 11 for FDA5, FDA5E, and FDA5L. The severity of change *n*_*T*_ is set to be 10, noted that our proposed algorithm is suitable for solving the problems with big *n*_*T*_. The frequency of change *τ*_*T*_ is set to be 10, 15, 20, 25, or 30 generations.

To detect environmental changes, 10 randomly selected individuals are recalculated at the beginning of each generation, and the algorithm will stop after detecting 10 environmental changes.

The population size for the four algorithms is 100; the offspring parameters in (10) of DMEA/PRI framework, that is, the crossover probability and mutation probability, are set to 0.8 and 0.08, respectively, and the variance of ADLM is set to 0.2. It is worth mentioning that 50% of population members are replaced with randomly selected predicted solutions to generate an initial population when a change occurs.

**Figure 1:**
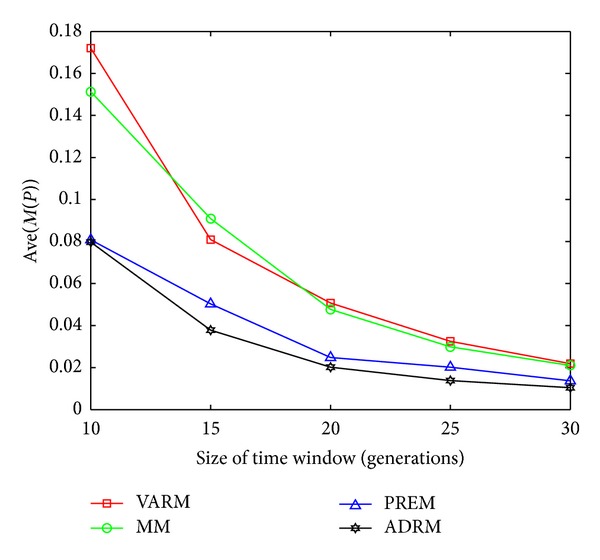
The statistical results of Ave(*M*(*P*)) for four prediction models on FDA1.

**Figure 2:**
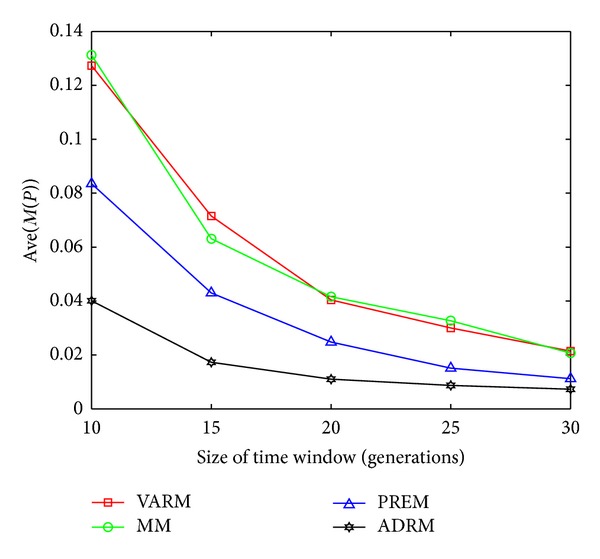
The statistical results of Ave(*M*(*P*)) for four prediction models on FDA1E.

**Figure 3:**
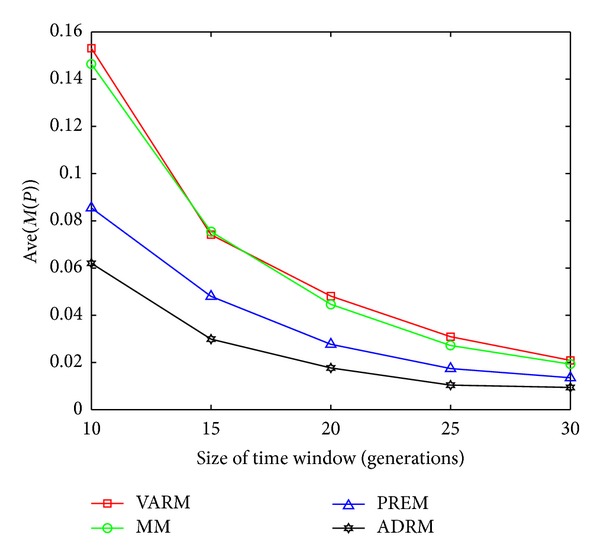
The statistical results of Ave(*M*(*P*)) for four prediction models on FDA1L.

**Figure 4:**
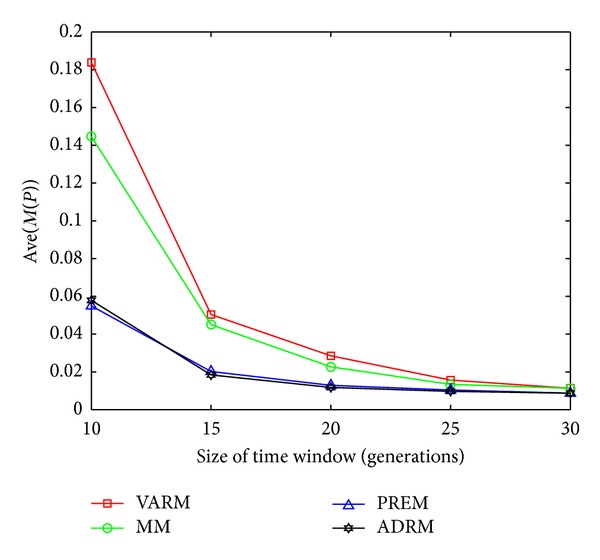
The statistical results of Ave(*M*(*P*)) for four prediction models on FDA5.

**Figure 5:**
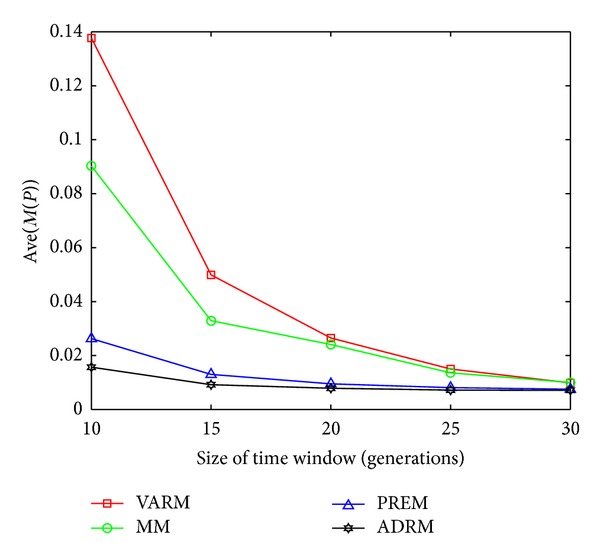
The statistical results of Ave(*M*(*P*)) for four prediction models on FDA5E.

**Figure 6:**
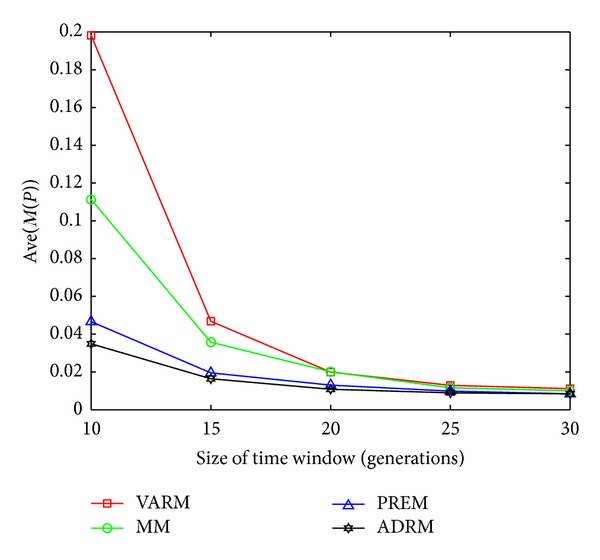
The statistical results of Ave(*M*(*P*)) for four prediction models on FDA5L.

The statistical results on FDA1, FDA1E, FDA1L, FDA5, FDA5E, and FDA5L with Ave(*M*(*P*)) indicators are shown in Figures 1, 2, 3, 4, 5, and 6.

In this experiment, the horizontal and the vertical axis of all figures are *τ*_*T*_ and the performance indicator Ave(*M*(*P*)), respectively. Based on this experiment, the following conclusions can be drawn through the comparative analysis of Figures 1–6:

1. The Ave(*M*(*P*)) value of any model in all figures decreases while *τ*_*T*_ increases from 10 to 30. The reasons for this conclusion could be stated as follows. NSGA-II is a very classic and effective Multiobjective evolutionary algorithm (MOEA); it is able to find the new PS after environmental change as long as there is enough time (generations) for NSGA-II to search for the new PS. Moreover, the increment of *τ*_*T*_ from 10 to 30 means that NSGA-II gets more time to look for the new PS. Therefore, the Ave(*M*(*P*)) value of all models decreases while *τ*_*T*_ increases from 10 to 30.
2. From the convergence point of view, in all figures, ADLM is better than PREM, PREM is better than MM, and MM is better than VARM. The reasons for this observation could be listed as follows. First, MM does not use historical information its randomness is strong and its convergence performance is weak, while VARM makes use of the historical information in the last two periods. However, VARM is not accurate enough to solve DMOP-TPS; therefore, the performance of MM is better than VARM. Second, compared to VARM and MM, PREM has plus the difference between two PSs in the last two periods, respectively, which guarantees the rationality and accuracy of the PREM on DMOP-TPS; therefore, the performance of PREM is better than that of MM and VARM. Last, ADLM makes use of the historical information in the last three periods, which is more abundant than the historical information used by any other models. Furthermore, ADLM, based on the analysis of mathematical properties of DMOP-TPS, is consistent with the dynamic translating law of DMOP-TPS. Thus ADLM can make the individuals predicted by itself very close to the new real PS. Therefore, the performance of ADLM is better than the other three prediction models.
3. The performance of ADLM on FAD1E (FAD5E) is the best in all figures. The reasons for this observation could be explained as follows. The PS of FAD1E (FAD5E) translates in a way of exponential function *f*(*t*) = 2^*t*^ − 1, *t* = (1/*n*_*T*_)⌊*τ*/*τ*_*T*_⌋, whose value changes very fiercely with time *t*, and ADLM simulates the translation tendency of the exponential function better than the other three models; therefore, the performance of ADLM is far better than that of the other three models on FAD1E (FAD5E).
4. The series of FAD1 problems are more difficult to be optimized than the series of FAD5 problems. In Figures 1–6, obviously, when *τ*_*T*_ is larger than 10, most of values of Ave(*M*(*P*)) in Figures 1–3 are larger than these in Figures 4–6; this is because the number of dimensions of decision variables is 30 for FDA1, FDA1E, and FDA1L and 11 for FDA5, FDA5E, and FDA5L; that is, the number of dimensions of the first three test problems is far bigger than that of the second three test problems; therefore, the series of FDA5 problems are easier to be optimized than the series of FDA1 problems.

## 6. Comparison of Time Complexity

In this section, the time complexity of the four prediction models will be analyzed and compared.

Generally, when designing one prediction model we need to consider not only the rationality of the prediction model but also the time complexity of the prediction model; that is because the time complexity is also an important measure for the performance of a prediction model.

Assuming that the population size is *N*, the dimension of the individual (decision vector) is *m*, and then the time complexity analysis is listed as follows.

1. The time complexity analysis of MM: because what MM needs to do is just to mutate every dimension of all individuals in current population without the use of historical information, so its time complexity is *O*(*mN*).
2. The time complexity analysis of VARM: VARM uses the historical information in the last two periods to predict new individuals. There are two steps for VARM to predict new individuals: first, finding *X*_*t*_ ∈ *Q*_*t*_ and corresponding *X*_*t*−1_ ∈ *Q*_*t*−1_ according to (16); second, updating every dimension of all individuals in current population according to the model of VARM. The time complexities of first and second step are *O*(*mN* ^2^) and *O*(*mN*), respectively; therefore, the total time complexity of VARM is *O*(*mN* ^2^).
3. The time complexity analysis of PREM: the steps of PREM are very similar with those of VARM when predicting new individuals, so the time complexity of PREM is *O*(*mN* ^2^).
4. The time complexity analysis of ADLM: ADLM uses the historical information in the last three periods to predict new individuals. There are two main steps for ADLM to predict new individuals: first, computing $\bar{X_{t}}-\bar{X_{t-1}}$ and corresponding $\bar{X_{t-1}}-\bar{X_{t-2}}$; second, updating every dimension of all individuals in current population according to the model of ADLM. The time complexities of first and second step are both *O*(*mN*); therefore, the total time complexity of ADLM is *O*(*mN*).

From the above analysis on the time complexity of four prediction models, it can be seen that the time complexity of ADLM and MM is the same and the time complexity of PREM and VARM is also the same. On the contrary, the time complexity of ADLM and MM is lower than that of PREM and VARM; therefore, from the convergence and time complexity point of view, ADLM outperform other prediction models when solving DMOP-TPS.

## 7. Conclusions

In this paper, we define a Multiobjective problem with translational Paretooptimal set (DMOP-TPS), and then we propose a new prediction model named ADLM to address DMOP-TPS. We have tested the proposed prediction model (ADLM) and compared it with other three superior prediction models on several DMOP-TPS benchmarks; furthermore, we analyzed the time complexity of the four prediction models.

From the simulation results on several DMOP-TPS test problems, four conclusions could be drew: (1) the Ave(*M*(*P*)) value of all models gets smaller while *τ*_*T*_ increases from 10 to 30; (2) from the convergence point of view ADLM is better than PREM, PREM is better than MM, and MM is better than VARM; (3) the performance of ADLM on FDA1E (FDA5E) outperforms other models; (4) the series of problems FDA1 are more difficult to be optimized than the series of problems FDA5. In addition, from the time complexity analysis of the four prediction models, it is clear that the time complexity of ADLM is very low.

Although a rich work has been made in this paper, the research on dynamic Multiobjective optimization is still in its very infancy and our work presented in this paper is also rather preliminary. More work remains to be done in the future; for example, it is important not only to detect the environmental changes but also to estimate the severity of changes in objective space; moreover, more appropriate variance selection for ADLM needs to be found, designing dedicated diversity controller by taking into account the problem structure, testing the suggested method on more benchmarks, and comparing it with other methods.

## Figures and Tables

**Algorithm 1:**
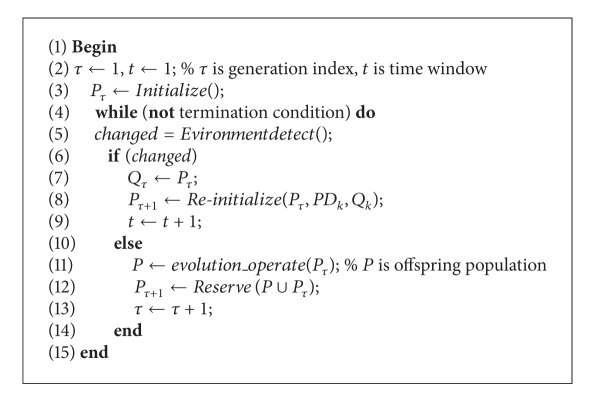
Dynamic multiobjective evolutionary algorithm with prediction model.

## Achnowledgments

This work was supported by the National Natural Science Foundation of China (Grant no. 61173107), National High technology Research and Development Program (863) (Grant no. 2012AA01A301-01), the Research Foundation in Industry Education Research Cooperation, Guangdong, Ministry of Education and Ministry of Science and Technology, China (Grant no. 2011A091000027), and the Research Foundation of Industry Education Research Cooperation of Huizhou, Guangdong (Grant no. 2012C050012012).
